# Dataset of establishment of gut microbiota: Molecular analysis of a cohort of 29 preterm Moroccan newborns

**DOI:** 10.1016/j.dib.2024.110129

**Published:** 2024-02-08

**Authors:** Kenza Hattoufi, Fatiha Raji, Houssain Tligui, Jaafar Heikel, Hassan Aguenaou, Amina Barkat

**Affiliations:** a National Reference Center in Neonatology and Nutrition, Children's Hospital, Ibn Sina University Hospital Centre, Rabat, Morocco; b Research Team on Health and Nutrition of Mother and Child, Faculty of Medicine and Pharmacy, Mohammed V University in Rabat, Morocco; c Joint Research Unit in Nutrition and Food, RDC-Nutrition AFRA/IAEA, Ibn Tofail University-CNESTEN, Morocco; d Research Laboratory of Ibn Sina University Hospital, Rabat, Morocco; e Faculty of Medicine and Pharmacy, Mohammed V University in Rabat, Morocco; f Mohammed VI University of Health Sciences, Casablanca, Morocco

**Keywords:** Gut Microbiota, Prematurity, Taqman, Syber Green, Morocco

## Abstract

The initial colonization of the intestine represents one of the most profound immunological exposures faced by the newborn. During the first three years of life, the intestinal microbial composition undergoes significant changes. At birth, the digestive tract is rapidly colonized by microorganisms of maternal and environmental origins. Microbiota's composition is influenced by various factors, including the mode of delivery, gestational age, type of feeding, and medication use. Through the current study, we specifically focused on elucidating the dynamics of gut microbiota colonization within the first three weeks of life of infants, shedding light on this critical phase of development.

A prospective cohort study involving 29 preterm infants was conducted from January to September 2021 at the National Reference Center for Neonatology and Nutrition, in collaboration with the research laboratory of Children's Hospital at the University Hospital Center Ibn Sina in Rabat. Stool samples were collected from each infant's diapers into a sterile tube and send for laboratory analysis.

A total of 203 stool samples were collected. For each newborn, one stool sample was obtained within the first 48 h after birth, followed by two samples per week over a period of three weeks. The microbial compositions of these samples were analyzed using real-time polymerase chain reaction.

Specifications Table

**Table:** 

Subject	Perinatology, Paediatrics and Child Health
Specific subject area	Microbiology, Pediatrics, molecular biology
Type of data	Excel file
How the data were acquired	All patient data were obtained from both the medical records and from the interviews conducted with the newborns' parents. The microbial compositions of the stool samples were analyzed using real-time polymerase chain reaction.
Data format	Tables in raw format (XLSX file)
Description of data collection	The stool samples were collected using sterile spatulas to retrieve samples from infant diapers. A stool sample was taken from each newborn at the first bowel movement on admission, then twice a week for a period of three weeks. DNA was extracted from the stool samples via Monarch® Genomic DNA Purification kit. Then, the samples were analyzed by using real-time polymerase chain reaction (RT-PCR). The screening targeted four phyla: Firmicutes, Bacteroidetes, Actinobacteria, and Proteobacteria.
Data source location	Institutions: National Reference Center in Neonatology and Nutrition, Children's Hospital, Ibn Sina University Hospital Center, Rabat, Morocco and Medical Research Laboratory, Children's Hospital, Ibn Sina University Hospital Center, Rabat, Morocco. City: Rabat Country: Morocco
Data accessibility	Repository name: Mendeley Data Data identification number: 10.17632/j2rnbkc2ct.1 Direct URL to data: https://data.mendeley.com/datasets/j2rnbkc2ct/1

## 1 Value of the Data

• This pioneering study was conducted in Morocco, generating a comprehensive dataset that encompasses sociodemographic, obstetrical, clinical, and paraclinical information. In addition, it resulted in identification of nine bacteria within the gut microbiota (GM) and the evolution of newborns during their hospitalization. This robust dataset holds significant potential for shaping the trajectory of future research in the field of microbiota, because it provides a solid foundation for in-depth analyses and investigations.
• Gynaecologists and paediatricians stand to gain valuable insights from this dataset to advocate for specific practices that contribute to optimal infant health. The data underscores the importance of promoting vaginal deliveries, emphasizing the benefits of breastfeeding, and guiding the judicious use of antibiotics. Armed with this knowledge, healthcare professionals can develop informed strategies to enhance the well-being of both mothers and newborns, in line with evidence-based practices for improved outcomes.
• The dataset serves as a rich resource for other researchers in understanding how GM varies across different populations. By analyzing sociodemographic information alongside microbiota data, scientists can identify patterns, correlations, and unique factors impacting distinct groups. This exploration not only enhances our understanding of the microbiota but also contributes to more personalized approaches in healthcare, which account for the diversity present in global populations.

## 2 Objective

A newborn's sterile gut undergoes progressive colonization by a diverse group of microorganisms, influenced by factors such as delivery mode (vaginal versus cesarean), maternal microbiomes, feeding practices (breast milk versus formula milk), environmental exposure, and antibiotic use [[1], [2]]. The establishment of GM significantly contributes to the development and maturation of the immune system, nutrient metabolism, and overall gut function [3]. An in-depth study of factors influencing the establishment of GM provides essential insights to promote optimal health outcomes and prevent potential imbalances that may contribute to subsequent health problems. This dataset specifically focuses on intestinal colonization by microbiota in premature newborns during the first three weeks of life.

## 3 Data Description

The establishment of GM in the early stages of life plays a crucial role in shaping a healthy and well-balanced individual. The human body harbors approximately 10–100 trillion microorganisms, mostly concentrated in the intestine due to its warmth, stability, and eutrophic environment [4,5].

The mechanism of intestinal colonization is a complex phenomenon. The gut microbiota is established immediately after the rupture of membranes. At birth, newborns are immersed in a rich and diverse bacterial environment. They are rapidly colonized by an initially simple microbiota primarily derived from the mother's microbiota [6,7]. They are then continuously exposed to other bacteria from the environment, nutrition, and adult skin bacteria. Consequently, there is a considerable interindividual variability in both the composition and patterns of bacterial colonization during the first weeks of life [8].

**Fig. 1:**
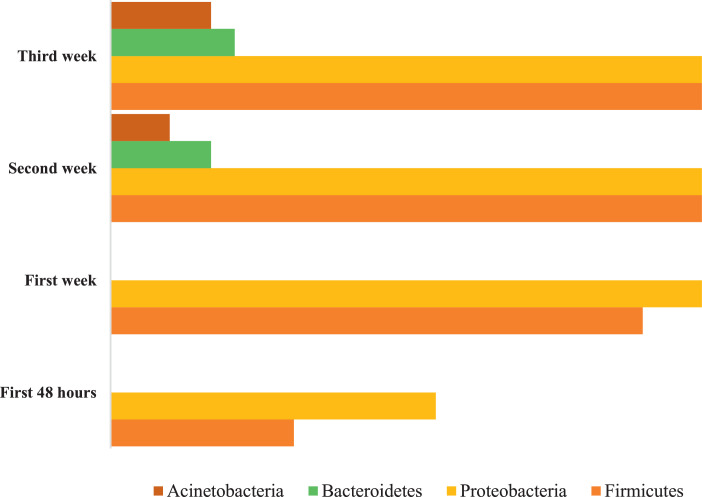
Profile of sample phyla.

The Excel file encompasses a wide range of data, including socio-demographic information such as maternal age, residence area, and consanguinity in addition to obstetrical data, such as parity, gravidity, pregnancy monitoring, and delivery mode. Newborn data include gender, infection risk factors, birth weight and height, and cranial perimeter. Additionally, the file incorporates clinical data, including clinical symptoms, as well as information on the patient's progress during hospitalization. The file also includes the results of RT-PCR testing of GM, with a focus on four phyla: Firmicutes, Bacteroidetes, Actinobacteria, and Proteobacteria (Fig. 1).

## 4 Experimental Design, Materials and Methods

### 4.1 Patients

Our study enrolled a total of 29 preterm infants [20 vaginally born; 9 cesarean-born], admitted to the Center for Neonatology and Nutrition and the Medical Research Laboratory at Children's Hospital in Rabat for prematurity management. The study was conducted from January to September 2021.

### 4.2 Inclusion criteria

• Preterm newborns, born between 28 and 36 gestational weeks, who received the standardized neonatal antibiotherapy according to the department's protocols.
• Newborns not requiring ventilatory support.
• Newborns aged 0–2 days.

### 4.3 Exclusion criteria

• Full-term newborns.
• Newborns presenting associated malformative pathology or morbidities at admission.
• Newborns who died during the study period.

### 4.4 Collected samples

**Fig. 2:**
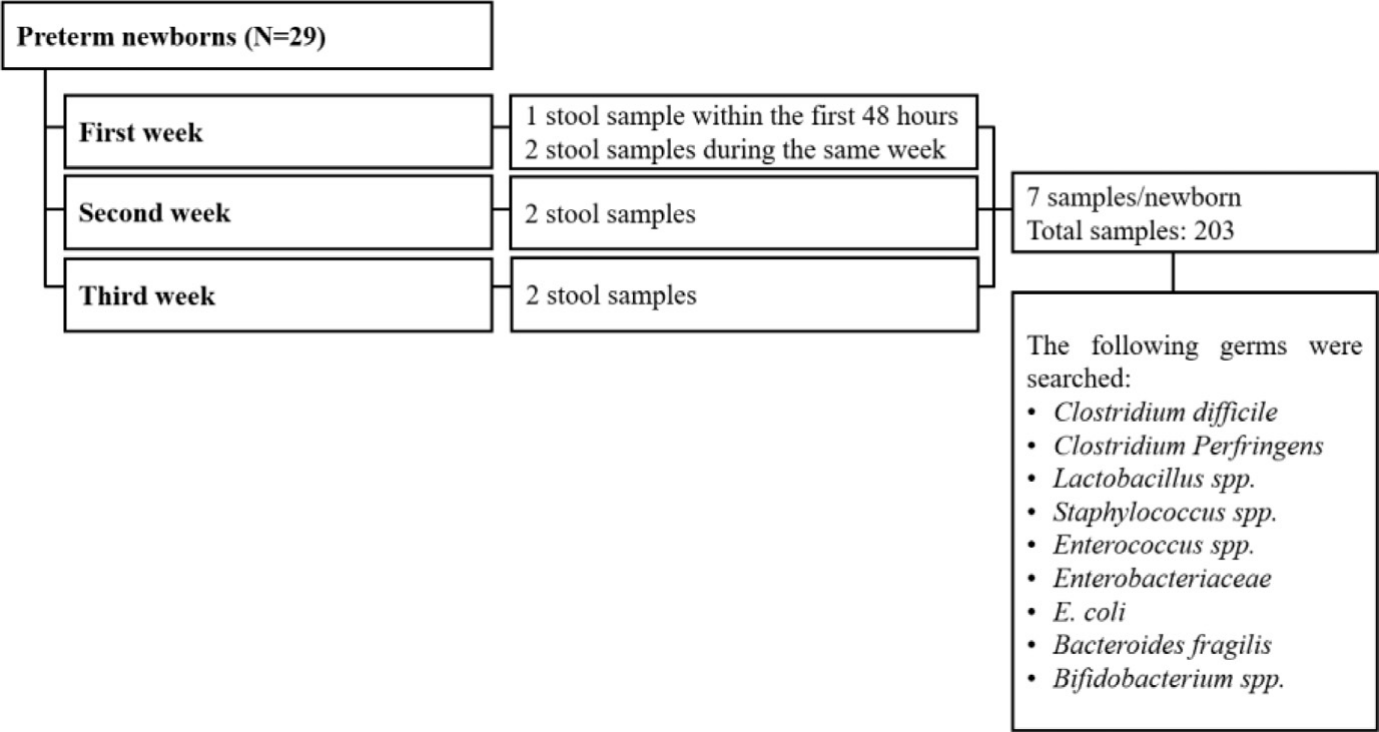
Flow chart of stool samples and germs studied.

Stool samples were collected from the infants' diapers then were initially stored at a temperature of 4 °C. Within 6 h, they were transferred to a sterile tube in the laboratory and subsequently stored at −80 °C for molecular studies. A total of seven stool samples were collected from each infant during the first three weeks after birth (Fig. 2).

### 4.5 Laboratory methods

After transfer and storage of the stool samples in the laboratory, DNA extraction was performed using the Monarch® Genomic DNA Purification Kit. Once this stage had been completed, we started the Real-Time Polymerase Chain Reaction (RT-PCR). Both Syber Green RT-PCR and Taqman RT-PCR methods were employed for this phase following the manufacturer's instructions.

The detection of Enterobactereaceae, Clostridium perfringens, Lactobacillus spp. and Staphylococcus spp. was performed using Syber Green method. While the detection of *Escherichia coli*, Clostridium difficile, Bifidobacterium spp., Enterococcus spp., and Bacteroides fragilis was carried out using Taqman RT-PCR method.

Target microorganisms were amplified using primers based on previous studies. The DNA primers utilized in the RT-qPCR for target bacteria are detailed in Table 1.

• Taqman method

**Table 1:** Primers sequences.

Phylum	Organism	Primer name	Primer sequences
Firmicutes	*Clostridium difficile*	Forward primer	TTG AGC GAT TTA CTT CGG TAA AGA
		Reverse primer	TGT ACT GGC TCA CCT TTG ATA TTC A
		Probe	CCA CGC GTT ACT CAC CCG TCC G
	*Clostridium perfringens*	*ClPer-1*	TAA CCT GCC TCA TAG AGT
		*ClPer-2*	TTT CAC ATC CCA CTT AAT C
	*Lactobacillus* spp.	Forward primer	AGC AGT AGG GAA TCT TCC A
		Reverse primer	CAC CGC TAC ACA TGG AG
	*Staphylococcus* spp.	Staph 758F	GAT GTG CGA AAG CGT GGG GAT
		Staph 1281R	GAA CTG AGA ACA ACT TTA TGG GA
	*Enterococcus* spp.	Forward primer	AGA AAT TCC AAA CGA ACT TG
		Reverse primer	CAG TGC TCT ACC TCC ATC ATT
		Probe	TGG TTC TCT CCG AAA TAG CTT TAG GGC TA
Proteobacteria	*Enterobacteriaceae*	En-lsu3F	TGC CGT AAC TTC GGG AGA AGG CA
		En-lsu3′R	TCA AGG CTC AAT GTT CAG TGTC
	*Escherichia coli*	Forward primer	CAT GCC GCG TGT ATG AAG AA
		Reverse primer	CGG GTA ACG TCA ATG AGC AAA
		Probe	TAT TAA CTT TAC TCC CTT CCT CCC CGC TGAA
Bacteroidetes	*Bacteroides fragilis*	Forward primer	CGG AGG ATC CGA GCG TTA
		Reverse primer	CCG CAA ACT TTC ACA ACT GAC TTA
		Probe	CGC TCC CTT TAA ACC CAA TAA ATC CGG
Acinetobacteria	*Bifidobacterium* spp.	Forward primer	GCG TGC TTA ACA CAT GCA AGT C
		Reverse primer	CAC CCG TTT CCA GGA GCT ATT
		Probe	TCA CGC ATT ACT CAC CCG TTC GCC

The RT-PCR was carried out using the Luna Universal Probe qPCR Master Mix, which is a 2x reaction mix optimized for RT- qPCR detection and quantification of target DNA sequences using hydrolysis probes.

Probe-based qPCR is particularly useful for determining DNA methylation status in specific regions. It uses real-time fluorescence released upon 5´→3´ exonuclease cleavage of a quenched, target-specific probe to measure DNA amplification at each cycle of a PCR. If the fluorescence signal is significantly detectable when compared with the background fluorescence, a quantification cycle or Cq value can be determined.

• Syber Green method

The RT-PCR was performed using the Luna Universal Probe qPCR Master Mix, which is 2X reaction mix for real-time qPCR detection and quantitation of target DNA sequences using the SYBR®/FAM channel of most real-time qPCR instruments.

Dye-based quantitative PCR (qPCR) uses real-time fluorescence of a double-stranded DNA (dsDNA) binding dye, most commonly SYBR® Green I, to measure DNA amplification during each cycle of a PCR. At a point where the fluorescence signal is confidently detected over the background fluorescence, a quantification cycle, or C_q_ value, can be determined.

## Ethics Statements

This study was conducted in accordance with the Declaration of Helsinki and was approved by the Ethics Committee of the Faculty of Medicine and Pharmacy of Rabat (Reference number CERB-01/20; date of approval, 16 November 2020).

All parents were clearly informed about the study objectives and received a summary of the protocol. Their participation in the study was conditional on signing the informed consent form.

## CRediT authorship contribution statement

**Kenza Hattoufi:** Conceptualization, Methodology, Formal analysis, Investigation, Writing – original draft. **Fatiha Raji:** Validation, Writing – review & editing. **Houssain Tligui:** Conceptualization, Investigation, Validation, Resources. **Jaafar Heikel:** Validation. **Hassan Aguenaou:** Validation, Writing – review & editing, Supervision. **Amina Barkat:** Conceptualization, Resources, Validation, Supervision, Writing – review & editing, Supervision.

## Data Availability

DATASET OF ESTABLISHMENT OF GUT MICROBIOTA: MOLECULAR ANALYSIS OF A COHORT OF 29 PRETERM MOROCCAN NEWBORNS (Original data) (Mendeley Data).
